# Antibiotic resistance, the 3As and the road ahead

**DOI:** 10.1186/s13099-018-0280-7

**Published:** 2018-12-22

**Authors:** Esther-Maria Antão, Szilvia Vincze, Regina Hanke, Lukas Klimmek, Katarzyna Suchecka, Antina Lübke-Becker, Lothar H. Wieler

**Affiliations:** 1 Centre for Infection Medicine, Institute of Microbiology and Epizootics, Freie University Berlin, Robert-von-Ostertag Str. 7-13, 14163 Berlin, Germany; 2 Robert-Koch-Institute, Nordufer 20, 13353 Berlin, Germany; 3 Lindgrün GmbH, Cuxhavener Str. 12, 10557 Berlin, Germany; 4 Department of Biological Safety, German Federal Institute for Risk Assessment, Max-Dohrn-Str. 8-10, 10589 Berlin, Germany

**Keywords:** Antibiotic resistance, One-Health, Awareness, Healthcare, Microbiome

## Abstract

Antibiotic resistance is by far one of the most important health threats of our time. Only a global concerted effort of several disciplines based on the One-Health concept will help in slowing down this process and potentially mitigate the ruin of healthcare we have come to enjoy. In this review, we attempt to summarize the most basic and important topics that serve as good information tools to create *Awareness*. The *Availability* of antibiotics or the lack thereof is another significant factor that must be given thought, and finally because antibiotic resistance is a problem that will not go away, it is important to have *Alternatives*. Together, we have the 3As, essential concepts, in dealing with this growing and complex problem.

## Background

### Antibiotics—a medical blessing

Antibiotics changed the face of medical practice. What was perceived as the greatest weapon in fighting infection yet, soon initiated a series of unstoppable events, the brunt of which we bear today: antibiotic resistance. Each time antibiotics are administered sensitive bacteria are slowly wiped out and resistant bacteria thrive. Often, antibiotics are still prescribed carelessly and sold without a prescription, by health professionals in many parts of the world [1]. Moreover, they are not just restricted to the treatment of humans but are also widely used in veterinary medicine. It is currently estimated that every year around 700,000 deaths are associated with drug resistance globally [2]. More people die from drug resistant infections than they do from measles and rabies put together (ca. 190,000 deaths globally) [3]. In Germany alone, around 400,000–600,000 people develop a nosocomial infection annually, of which 30,000–35,000 are caused by a multidrug-resistant organism [4] and it is estimated that the number of patient deaths due to multidrug-resistance lie between 1000 and 4000 [4]. A study analyzing the resistance situation in Germany showed that MRSA-resistance rates and the prevalence of methicillin-resistant *Staphylococcus aureus* (MRSA) have remained at a stable level over the last few years, however, vancomycin-resistant enterococci (VRE) and multidrug-resistant Gram-negative (MRGN) bacteria have increased considerably [5]. There also is a rising tendency in the presence of carbapenem-resistant *Klebsiella pneumoniae* isolates, as published by the antibiotic resistance surveillance (ARS) of the Robert Koch Institute, the national health institute in Germany (ars.rki.de) [6].

Antibiotic resistance threatens global public health, and the judicious use of antibiotics is required more than ever before. The key lies in the ‘One-Health’ approach which necessitates a collaboration between the human and veterinary medical sectors to better understand the relationship between humans, animals and their environment with respect to their health.

## Awareness

### Antibiotic resistance and the One-Health concept

**Fig. 1:**
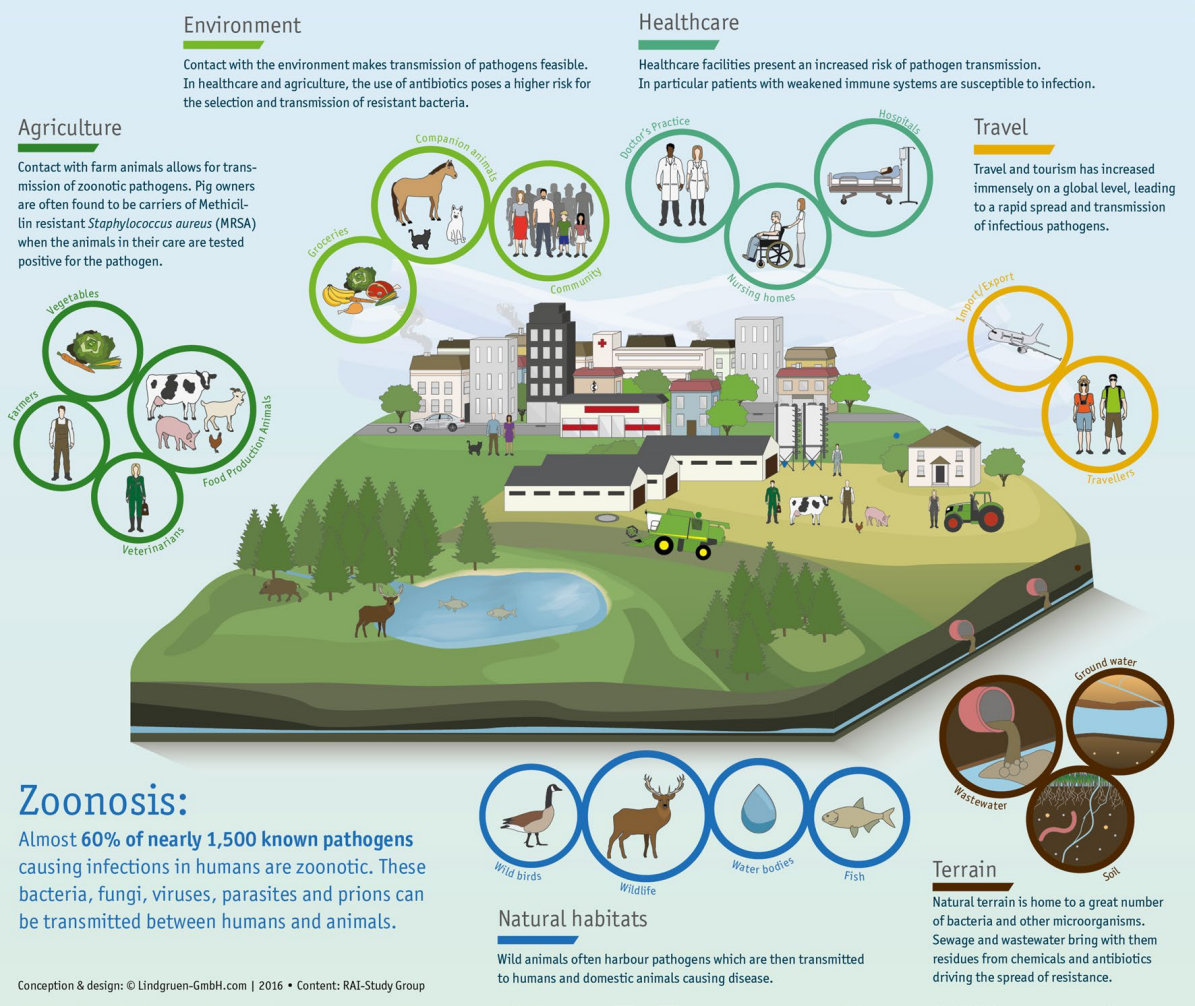
Infographic about the One-Health Concept considering all aspects of life

It was the famous Rudolf Virchow, who made the link between infections in humans and animals, and coined the term zoonosis. His fascination for helminthology especially the life cycle of the nematode *Trichinella spiralis* inspired by his childhood spent watching butchers at work made him a prominent advocate of meat inspection, a factor vital to public health, though it was the renowned veterinarian Robert von Ostertag who eventually came up with the first Handbook of Meat Inspection several years later [7, 8]. Sir William Osler, father of modern medicine, backed this concept to a great extent, though it was Calvin Schwabe, a twentieth Century veterinarian, who is said to have come up with the expression One-Medicine and propagated its practice in modern times [9]. Today we know this concept as One-Health which recognizes that human health is linked with the health of animals and the environment, and only a concerted effort of multiple disciplines and the bridging of the two professions (human and veterinary medicine) globally can achieve optimal health for humans and animals (Fig. 1). Zoonotic diseases are those that can be transferred between humans and animals. Over 60% of all known pathogens that infect humans are zoonotic in nature [10, 11].

*Escherichia coli* is a bacterium that makes up part of the intestinal microbiota in both humans and animals. Given the right setting, however, these bugs can often cause mild to very severe infections, posing a problem for both human and veterinary medicine. Antibiotics have, however, now lost the power to treat many *E. coli* infections, the most common of these being those caused by extended spectrum β-lactamase (ESBL) producing *E. coli*, which are resistant to several β-lactam antibiotics, including the penicillins and cephalosporins and which are now widespread among both humans and animals [11, 12].

The appearance of genetically similar drug-resistant strains across several species is a further cause for worry. In 2014 Ewers et al. published the results of a study, which identified a novel genotype among certain ST648 extraintestinal pathogenic *E. coli* (ExPEC) subgroups of the phylogenetic lineage D, isolated from animals. This genotype combined both multiresistance (CTX-M) and extraintestinal virulence [13]. It was interesting to find the CTX-M-15 genotype, a human linked β-lactamase type prevalent among companion animal isolates. The study concluded that non B2 lineages were relevant to both humans and animals and that ST648 strains were potentially zoonotic. A year later, when analysing faeces from dogs within a clinical and non-clinical setting, Schaufler et al. found high rates of ESBL producing *E. coli* shed by dogs outside the clinical setting. What was more alarming was that when comparing strains from dogs in a clinical and non-clinical setting, several strains were found to be clonal [14]. Such findings started to become a regular occurrence. In 2016, Schaufler et al. once again provided evidence for an interspecies transmission of a new successful ESBL producing *E. coli* clone (ST410) between wildlife, humans, companion animals and the environment [15]. Meanwhile McNally et al., when studying the ecology of another multidrug-resistant *E. coli* lineage ST131, which is pandemic, looked at it from a different perspective of lineage evolution that combines core, accessory and gene regulatory region genome analysis. They found that human, dog, cat and wild bird isolates can move freely across niches, without any obvious signals of ecological adaptation and niche segregation, once again providing evidence for the zoonotic nature of this clone [16]. In a separate study Günther et al. emphasized the global environmental dimension and zoonotic character of ESBL producing *E. coli*, after finding similarities between Mongolian wildlife isolates and European clinical isolates, and observing that environmental isolates harbour stably integrated, chromosomally encoded resistance factors [17], while yet another study revealed that green sea turtles might be harbouring and spreading superbugs near the Great Barrier Reef. *Enterobacteriales* were isolated from cloacal swabs of captured green sea turtles, and found to be resistant to twelve different antibiotics from six different classes [18]. Almost 40% of the isolated *Enterobacteriales* were found to be multidrug-resistant.

With global travel surging, the crowded skies are not just a worry for aviation agencies and air traffic controllers, but for public health experts alike. In 2015 Lübbert et al. examined stool samples from travellers returning to Germany and found that 30% were colonized by ESBL *E. coli* following their travel, with those travelling back from India showing the highest colonization levels of ESBL producing *Enterobacteriaceae* (73%) [19]. This was confirmed in 2016, when it was reported that travellers returning from India and Southeast Asia were a relevant source of spread of multidrug-resistant *E. coli* [20]. Looking at it from a different perspective Bengtsson-Palme et al. examined stool isolates of Swedish students on exchange programs in the Indian peninsula and central Africa. They concluded that the human microbiome acts as a vehicle for antibiotic resistance genes, while maintaining a stable taxonomic diversity [21].

The worldwide prevalence of an important drug-resistant pathogen *methicillin*-resistant *Staphylococcus aureus* (MRSA) is well documented. Hospital-acquired (HA), Community-acquired (CA) and Livestock-associated (LA) MRSA are the most common terms used to categorize the incidence of MRSA. Cuny et al. reported in 2015 that 10% of the MRSA isolated from humans were in fact LA-MRSA [22]. In addition, the study showed, that 77–88% of humans with exposure to pigs on farms were likely to show nasal colonization of MRSA, of which transfer to family members occurred in 4–5% of these cases. This suggests that pig farmers can be easily colonized by MRSA which therefore places them in a risk category in the recommendations for the prevention and control of MRSA. While being colonized by MRSA is no cause for worry, certain predisposing conditions like a weakened immune system or invasive surgeries could make it conducive for severe infection which is then difficult to treat.

Close contact with animals whether based on occupation (farming industry) or simply by having pets at home, provides many opportunities for pathogens, including drug-resistant bacteria to be exchanged between humans and animals [23]. Walther et al. described this when they found pets and their owners colonized by *Staphylococcus pseudintermedius* in a study in 2012 [24]. They also reported a novel MRSA variant identified in companion animals, which was not restricted to humans or ruminants, therefore suggesting a potential zoonotic risk of this pathogen [25]. Additionally, Vincze et al. showed in separate studies that methicillin resistant and susceptible *S. aureus* strains from companion animal infections were highly similar to strains from human infections and colonization [26, 27]. These shared *S. aureus* populations serve as indicators for a potential exchange of strains between humans and companion animals. Previously, Paterson et al. were able to prove a direct MRSA transmission between infected dogs and colonized humans in both directions [28]. When analyzing the possibility of contamination of commercial poultry meat (broiler and free-range) with pathogenic or multidrug-resistant *E. coli* in retail chain poultry meat markets in India, Hussain et al. found a higher prevalence of ESBL—*E. coli* among broiler chickens, and confirmed two globally emergent human pathogenic lineages of *E. coli*, suggesting that poultry may be an indirect public health risk, by being a possible carrier of non-pathogenic multidrug-resistant *E. coli* as well as human *E. coli* pathotypes [29]. With all the evidence piling up, the need of the hour is dealing with this problem, which is only possible in a combined effort of interdisciplinary sectors modelled on the One-Health concept, to help slow down the spread of drug-resistance.

### What came first, antibiotics or resistance?

In 2011, a group of scientists dug up ancient DNA hidden and preserved below ground at Dawson City, Yukon, which lies on the Tintina Fault, with its subarctic climate, population of 1375 and underlain with permafrost. They reported targeted metagenomics analyses of rigorously authenticated DNA, obtained from 30,000-year-old Beringian permafrost sediments [30] and went on to identify a highly diverse collection of genes encoding resistance to ß-lactam, tetracycline and glycopeptide antibiotics. Previously, the same group suggested that soil could serve as an under recognized reservoir for resistance that has already emerged or has the potential to emerge in clinically important bacteria [31]. Their research findings are proof of antibiotic resistance being a natural phenomenon. This is significant when human and veterinary medical sectors work together using a One-Health approach. In a study in Germany, GPs, hospital physicians, pig farmers, veterinarians and members of the general public were surveyed to understand the perceptions and attitudes towards antibiotic resistance. Each group tended to identify drivers of antibiotic resistance as being from outside their own area of activity [32]. When talking about the problem of resistance to antibiotics, playing the blame game is futile, because antibiotic resistance does eventually find its way into the ecosystem.

**Fig. 2:**
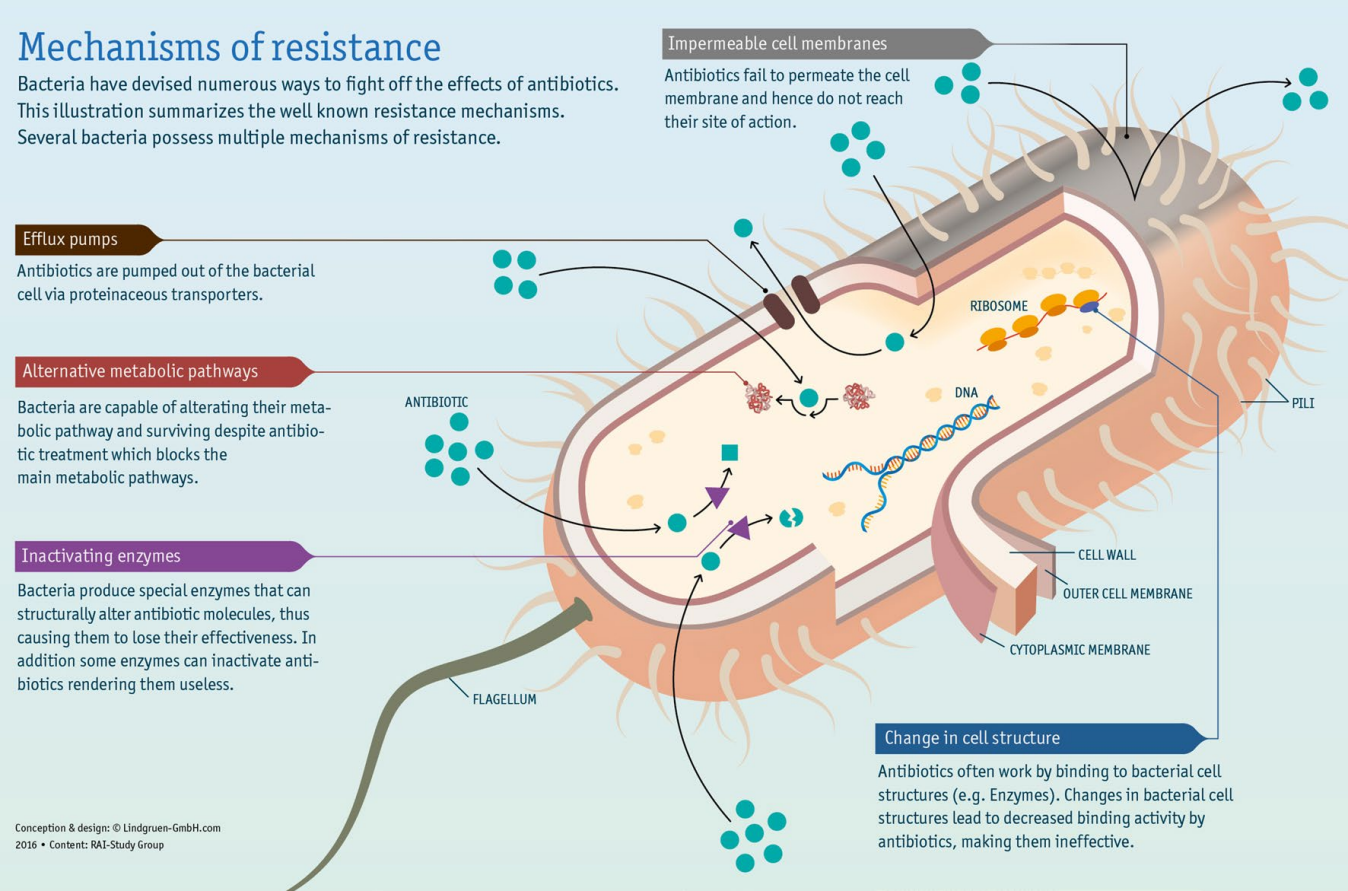
Infographic about the mechanisms of antibiotic resistance in bacteria

To understand this, one must once again go back to the fundamentals of microbiology. There are several mechanisms by which an organism becomes resistant to antibiotics (Fig. 2), but a distinction is to be made between natural (intrinsic) resistance and acquired resistance [33]. Natural resistance describes the intrinsic properties of a bacterium which do not permit an antibiotic to work. Acquired resistance on the other hand describes the resistance of a bacterium to an antibiotic to which it was previously susceptible. Additionally, bacteria can become resistant through the acquisition of extrachromosomal elements or foreign DNA carrying information for antibiotic resistance, the best example being, colistin resistance, transferred by the *mcr* gene harboured on a plasmid [34]. This kind of resistance is crucial, as it can spread easily between and across bacterial species.

### Antibiotics: knowing when, knowing how

Sometimes, choosing and prescribing antibiotics can be quite a predicament for professionals. As a start, the prescriber needs to be certain that prescribing the antibiotic is the correct choice in that individual situation, in order to avoid unnecessary use of antibiotics. Following the initial decision, choosing the right antibiotic might be the next hurdle. Good antimicrobial stewardship is the rational and responsible use of antibiotics and focusses on the choice of antibiotic, therapy duration and dose, route of administration, as well as evidence of a bacterial infection [35]. The aim of antimicrobial stewardship is to obtain the best clinical outcome and reduce toxicity for the patient, while simultaneously ensuring reduced selection and resistance [35, 36]. Antimicrobial stewardship programmes are important in hospitals and clinics, with infectious disease specialists, clinical microbiologists and clinical pharmacists working together to constitute the core of such programmes promoting responsible antibiotic use [37], though such programmes are not implemented everywhere. Medical professionals must be given appropriate training in good antimicrobial stewardship in order to encourage rational prescribing of antibiotics in private practices and outpatient clinics. With successful antimicrobial stewardship programmes and trainings in place, a significant contribution can be made towards reducing and slowing down the development and spread of resistance.

**Fig. 3:**
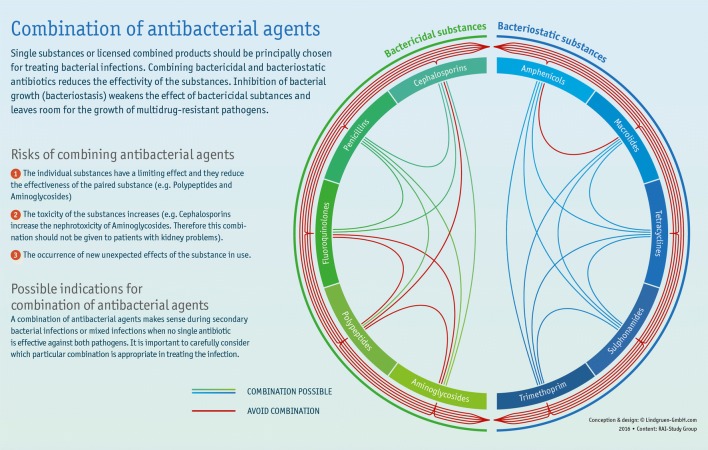
Infographic depicting the possibilities of combining antibacterial agents

Antimicrobial stewardship programmes can extend beyond human medicine into the veterinary sector. For example, when selecting an appropriate antibiotic for treating infections in farm animals, one must consider many factors, including diagnosis, route of administration, duration of therapy, spectrum of activity, herd-specific resistance problems, type of activity (Bacteriostatic vs. Bactericidal) and pharmacokinetics (Fig. 3), much like it ought to be done in human medicine. In addition, antibiotics must be approved for the specific treatment, and “Reserve antibiotics” must be thought about very carefully before being selected. When treating bacterial infections, using single antimicrobials (monotherapy) over combinations might be a good idea. A combination of bacteriostatic and bactericidal antibiotics could result in reduced effectivity against the pathogen being treated [38–40]. In the event of a secondary bacterial infection or mixed infections a combination therapy only makes sense if no antibiotic can be found that would effectively kill every pathogen in question. This applies to both human and veterinary medicine.

The World Health Organisation (WHO) very recently updated their ‘Essential Medicines List’ with new advice on the use of antibiotics. Experts have placed antibiotics into three categories—Access, Watch and Reserve—with recommendations on when these should and can be used [41]. The purpose of the new recommendation is to ensure that antibiotics are available to everyone when they are absolutely required, infection-appropriate antibiotics are prescribed, development of antibiotic resistance is reduced and “last-resort” antibiotics are saved for a time when all other antibiotics fail.

To deal with one of the most complex problems affecting global health, one must therefore understand the problem in its entirety. Antibiotic resistance affects many spheres of life with many factors driving its spread. There is no easy way out; and so, we have first in foremost summarized the most basic and important topics that serve as good information tools to create *Awareness*, which can assist us in dealing with the problem of antibiotic resistance. In a One-Health scenario each one has their part to play and if done well, it stands to benefit every sector in question.

Awareness, however, also goes beyond basic knowledge of antibiotic resistance. A significant aspect here is an awareness about the problems of failing antibiotics and the consequences of untreatable infections which is crucial for the rational and appropriate use of antibiotics. Conversely the lack of awareness can create uncertainty and poor judgement in prescribers and patients alike. A recent study showed that patients who have used antibiotics might have more knowledge as a result of them having to deal with the topic of antibiotic use, and the authors suggest that health literacy could be a preventive mechanism with regard to using antibiotics critically [42]. Worldwide, educational programmes and campaigns are being successfully implemented in an effort to create awareness not just among professionals, but among the general public as well [43–46]. In Germany, much is being done in an effort to deal with the problem of antibiotic resistance. Priorities for antibiotic resistance prevention are set at different levels: nationally with the German Antimicrobial Resistance Strategy (DART2008, updated in 2015) and regionally by networks created in 2004 with support from the National Public Health Institute, the Robert Koch Institute [47]. The 2015 German national action plan on drug resistance, DART2020, aims at implementing and strengthening the One-Health concept nationally and internationally, detect early resistance development, improve therapeutic options, prevent infections, create awareness, as well as support further research that would be beneficial in the fight against resistance [48]. A good example of one such research projects, funded by the Federal Ministry of Education and Research (BMBF), is *InfectControl 2020*: a consortium of academics and business partners coming together to find solutions on a national level. For the first time in Germany, a pilot project of *InfectControl* 2020, *Rational Antibiotic Use* via *Information and Communication (RAI)*, which is modelled on the One-Health concept, has brought together stakeholders from various areas of human and veterinary medical sciences as well as the communication sciences to address this issue [49]. The RAI-project (http://www.rai-projekt.de) aims at promoting rational antibiotic use in veterinary medicine, in particular, pig farming, as well as in human medicine, surgery and intensive care units, travel medicine, and primary care, primarily through awareness and intervention strategies [32, 50].

## Availability

The availability of antibiotics or the lack thereof, is an element of antibiotic resistance which must be given careful thought. The discrepancy in the availability of antibiotics in developed and developing countries suggests a need for change in attitudes and policies. The costs of not addressing the rise in antimicrobial resistance could lead to an annual reduction in gross domestic product by 3–8% by the year 2050 with low- and middle-income countries being hit the hardest [51]. In many developing countries, particularly in rural areas, patients sometimes have no access to medical personnel, let alone antibiotics. Meanwhile in larger towns and cities of these same countries, antibiotics are sold over the counter to those who can afford them even without a prescription from a healthcare professional [1]. In developed countries, there ends up being a surplus of available antibiotics, especially when administered unjustifiably [52]. In Germany, the total antibiotic consumption in human medicine is around 800 tons, of which 600 tons are used in outpatient care, more than half of which are prescribed by GPs [50]. Though Germany is one those countries with a lower level of antibiotic consumption, the proportion of reserve antibiotics used is still high [50]. There is a need to balance access to essential medications, particularly in low- and middle-income countries where the burden of infectious diseases still outweighs the burden of resistant infections [53]. While we go about saving the antibiotics that we still have through good stewardship practices, many ethical questions must be asked, when looking at this problem from the perspective of a patient who has no hope of recovery from treatable infections, with no access to good healthcare and antibiotics. Besides this, we face the problem of a lack of availability of new antibiotics for several reasons, which cannot be dealt with in detail within the scope of this review. This aspect therefore needs to be tackled at the policy level especially when implementing regulations regarding the availability, use and sale of antibiotics. Rochford et al. have rightly proposed an international agreement to ensure that antibiotics are available for future generations [51].

## Alternatives

Because antibiotic resistance is a problem that will not go away, it is important to have other alternatives. With this in mind it is essential, that the alternatives we look for are not necessarily substances that might replace the function of antibiotics per se, that is fight off infection, but methods and technologies that can support medicine in strengthening the immune system, besides preventing and treating infections.

Bacteriophages were used in 1917 by their discoverer Felix d’Herelle to successfully treat bacterial infections [54]. Due to the success with antibiotics later discovered, bacteriophages were set aside, but very recently researchers and doctors at the University of California San Diego School of Medicine used novel phage therapy to treat a patient near death, suffering from an infection with a multidrug-resistant strain of *Acinetobacter baumannii*, thus saving his life [55]. CRISPR technology is also being reviewed as an option to treat resistant infections [56, 57]. In 2016, Zipperer et al. discovered that a human commensal *Staphylococcus lugdunensis* produces lugdunin, a novel antibiotic, which shows bactericidal activity against many major harmful bacteria, including Methicillin-resistant *Staphylococcus aureus* (MRSA) and Vancomycin-resistant Enterococcus isolates, both of which are one of the major causes for concern for antibiotic resistance [58]. In 2018, Geldart et al. engineered the *E. coli* Nissle 1917 strain to produce and secrete antimicrobial peptides to specifically target and kill Enterococcus, with first results in a Vancomycin resistant Enterococcus (VRE) colonization mouse model appearing very promising [59].

We would like to focus on the human microbiome as a promising alternative with its immense scope and potential, as well as the fact that new research provides increasing evidence for links between the gut microbiome and health [60, 61]. The gut microbiota is known to play a role in anxiety, mood, cognition and pain which is exerted via the gut-brain axis and probiotics are frequently being used to treat a range of conditions including constipation, allergic reactions and infections [62]. In addition, the microbiome has already made headlines with a severe case of antibiotic resistance. A few years ago in a clinical trial, a faecal microbiota transplant (“Stool Transplant”) using purified intestinal bacterial cultures was successfully carried out in a patient suffering from a *Clostridium difficile* infection that had repeatedly failed standard antibiotics [63]. This is therefore a very promising method, given that in some cases it may be a last resort treatment option.

All of these promising technologies may not always be an easy solution, let alone feasible, especially in areas where access to basic healthcare and medicines is still a huge problem; however, these alternatives might soon be the only way forward. It may be a while before these methods become the new standard treatment, though from a global public health perspective, we need to take all these aspects into consideration when planning for a healthy future for every individual. Finally, if we do not find ways to tackle antibiotic resistance, we could find ourselves being afraid of a simple twig which could turn out to be a potentially deadly weapon.

## References

[1] Morgan DJ, Okeke IN, Laxminarayan R, Perencevich EN, Weisenberg S (2011). Non-prescription antimicrobial use worldwide: a systematic review. Lancet Infect Dis..

[2] European Centre of Disease Control (ECDC) (2016). Last-line antibiotics are failing: options to address this urgent threat to patients and healthcare systems.

[3] WHO (2017). Antimicrobial resistance fact sheet.

[4] Gastmeier P, Geffers C, Herrmann M, Lemmen S, Salzberger B, Seifert H (2016). Nosocomial infections and infections with multidrug-resistant pathogens—frequency and mortality. Dtsch Med Wochenschr.

[5] Maechler F, Geffers C, Schwab F, Pena Diaz LA, Behnke M, Gastmeier P (2017). Development of antimicrobial resistance in Germany: what is the current situation?. Med Klin Intensivmed Notfmed..

[6] Koppe U, von Laer A, Kroll LE, Noll I, Feig M, Schneider M (2018). Carbapenem non-susceptibility of Klebsiella pneumoniae isolates in hospitals from 2011 to 2016, data from the German Antimicrobial Resistance Surveillance (ARS). Antimicrob Resist Infect Control..

[7] Saunders LZ (2000). Virchow’s contributions to veterinary medicine: celebrated then, forgotten now. Vet Pathol.

[8] von Ostertag R (1904). Handbook of meat inspection.

[9] Cardiff RD, Ward JM, Barthold SW (2008). ‘One medicine—one pathology’: are veterinary and human pathology prepared?. Lab Invest.

[10] Cantas L, Suer K (2014). Review: the important bacterial zoonoses in “one health” concept. Front Public Health..

[11] Vincze S, Schneider S, Lübke-Becker A (2016). One-Health-Konzept—Zusammenhänge verstehen.

[12] Ewers C, Bethe A, Semmler T, Guenther S, Wieler LH (2012). Extended-spectrum beta-lactamase-producing and AmpC-producing *Escherichia coli* from livestock and companion animals, and their putative impact on public health: a global perspective. Clin Microbiol Infect.

[13] Ewers C, Bethe A, Stamm I, Grobbel M, Kopp PA, Guerra B (2014). CTX-M-15-D-ST648 *Escherichia coli* from companion animals and horses: another pandemic clone combining multiresistance and extraintestinal virulence?. J Antimicrob Chemother.

[14] Schaufler K, Bethe A, Lubke-Becker A, Ewers C, Kohn B, Wieler LH (2015). Putative connection between zoonotic multiresistant extended-spectrum beta-lactamase (ESBL)-producing *Escherichia coli* in dog feces from a veterinary campus and clinical isolates from dogs. Infect Ecol Epidemiol..

[15] Schaufler K, Semmler T, Wieler LH, Wohrmann M, Baddam R, Ahmed N (2016). Clonal spread and interspecies transmission of clinically relevant ESBL-producing *Escherichia coli* of ST410–another successful pandemic clone?. FEMS Microbiol Ecol..

[16] McNally A, Oren Y, Kelly D, Pascoe B, Dunn S, Sreecharan T (2016). Combined analysis of variation in core, accessory and regulatory genome regions provides a super-resolution view into the evolution of bacterial populations. PLoS Genet.

[17] Guenther S, Semmler T, Stubbe A, Stubbe M, Wieler LH, Schaufler K (2017). Chromosomally encoded ESBL genes in *Escherichia coli* of ST38 from Mongolian wild birds. J Antimicrob Chemother..

[18] Ahasan MS, Picard J, Elliott L, Kinobe R, Owens L, Ariel E (2017). Evidence of antibiotic resistance in Enterobacteriales isolated from green sea turtles, *Chelonia mydas* on the Great Barrier Reef. Mar Pollut Bull..

[19] Lubbert C, Straube L, Stein C, Makarewicz O, Schubert S, Mossner J (2015). Colonization with extended-spectrum beta-lactamase-producing and carbapenemase-producing Enterobacteriaceae in international travelers returning to Germany. Int J Med Microbiol.

[20] Barreto Miranda I, Ignatius R, Pfuller R, Friedrich-Janicke B, Steiner F, Paland M (2016). High carriage rate of ESBL-producing Enterobacteriaceae at presentation and follow-up among travellers with gastrointestinal complaints returning from India and Southeast Asia. J Travel Med..

[21] Bengtsson-Palme J, Angelin M, Huss M, Kjellqvist S, Kristiansson E, Palmgren H (2015). The human gut microbiome as a transporter of antibiotic resistance genes between continents. Antimicrob Agents Chemother.

[22] Cuny C, Wieler LH, Witte W (2015). Livestock-associated MRSA: the impact on humans. Antibiotics (Basel)..

[23] Walther B, Tedin K, Lubke-Becker A (2017). Multidrug-resistant opportunistic pathogens challenging veterinary infection control. Vet Microbiol.

[24] Walther B, Hermes J, Cuny C, Wieler LH, Vincze S, Abou Elnaga Y (2012). Sharing more than friendship–nasal colonization with coagulase-positive staphylococci (CPS) and co-habitation aspects of dogs and their owners. PLoS ONE.

[25] Walther B, Wieler LH, Vincze S, Antao EM, Brandenburg A, Stamm I (2012). MRSA variant in companion animals. Emerg Infect Dis.

[26] Vincze S, Stamm I, Monecke S, Kopp PA, Semmler T, Wieler LH (2013). Molecular analysis of human and canine *Staphylococcus aureus* strains reveals distinct extended-host-spectrum genotypes independent of their methicillin resistance. Appl Environ Microbiol.

[27] Vincze S, Stamm I, Kopp PA, Hermes J, Adlhoch C, Semmler T (2014). Alarming proportions of methicillin-resistant *Staphylococcus aureus* (MRSA) in wound samples from companion animals, Germany 2010–2012. PLoS ONE.

[28] Paterson GK, Harrison EM, Murray GG, Welch JJ, Warland JH, Holden MT (2015). Capturing the cloud of diversity reveals complexity and heterogeneity of MRSA carriage, infection and transmission. Nat Commun..

[29] Hussain A, Shaik S, Ranjan A, Nandanwar N, Tiwari SK, Majid M (2017). Risk of transmission of antimicrobial resistant *Escherichia coli* from commercial broiler and free-range retail chicken in India. Front Microbiol..

[30] D’Costa VM, King CE, Kalan L, Morar M, Sung WW, Schwarz C (2011). Antibiotic resistance is ancient. Nature.

[31] D’Costa VM, McGrann KM, Hughes DW, Wright GD (2006). Sampling the antibiotic resistome. Science.

[32] Schneider S, Salm F, Vincze S, Moeser A, Petruschke I, Schmucker K (2018). Perceptions and attitudes regarding antibiotic resistance in Germany: a cross-sectoral survey amongst physicians, veterinarians, farmers and the general public. J Antimicrob Chemother..

[33] Vincze S, Antão E, Lübke-Becker A (2017). Selektion und Resistenzmechanismen—Entstehung und Ausbreitung resistenter Bakterien.

[34] Malhotra-Kumar S, Xavier BB, Das AJ, Lammens C, Butaye P, Goossens H (2016). Colistin resistance gene mcr-1 harboured on a multidrug resistant plasmid. Lancet Infect Dis..

[35] Dyar OJ, Huttner B, Schouten J, Pulcini C, Esgap (2017). What is antimicrobial stewardship?. Clin Microbiol Infect..

[36] Pulcini C (2017). Antibiotic stewardship: a European perspective. FEMS Microbiol Lett..

[37] Pulcini C, Mainardi JL (2014). Antimicrobial stewardship: an international emergency. Clin Microbiol Infect.

[38] Vincze S, Lübke-Becker A (2016). Antibiotika: Gut zu wissen—grundlagen der pharmakologie.

[39] Ocampo PS, Lazar V, Papp B, Arnoldini M, AbelzurWiesch P, Busa-Fekete R (2014). Antagonism between bacteriostatic and bactericidal antibiotics is prevalent. Antimicrob Agents Chemother..

[40] Bollenbach T (2015). Antimicrobial interactions: mechanisms and implications for drug discovery and resistance evolution. Curr Opin Microbiol.

[41] WHO (2017). WHO updates Essential Medicines List with new advice on use of antibiotics, and adds medicines for hepatitis C, HIV, tuberculosis, and cancer [press release].

[42] Salm F, Ernsting C, Kuhlmey A, Kanzler M, Gastmeier P, Gellert P (2018). Antibiotic use, knowledge and health literacy among the general population in Berlin, Germany and its surrounding rural areas. PLoS ONE.

[43] Alberta Health Services and the British Columbia Centre for Disease Control (2015). Do bugs need drugs: a community program for wise use of antibiotics.

[44] Public Health England (2014). Antibiotic awareness: quizzes and crosswords.

[45] Ministry of Health and Animal Welfare, Government of India (2017). National Action Plan on Antimicrobial Resistance (NAP-AMR) 2017–2021.

[46] WHO (2017). World antibiotic awareness week.

[47] Birgand G, Castro-Sanchez E, Hansen S, Gastmeier P, Lucet JC, Ferlie E (2018). Comparison of governance approaches for the control of antimicrobial resistance: analysis of three European countries. Antimicrob Resist Infect Control..

[48] DART 2020 (2015). Antibiotika-Resistenzen bekämpfen zum Wohl von Mensch und Tier.

[49] Neue Antiinfektionsstrategien: Wissenschaft, Gesellschaft, Wirtschaft. Jena: Fördermaßnahme “Zwanzig20—Partnerschaft für Innovation” des Bundesministeriums für Bildung und Forschung (BMBF); 2014.

[50] Salm F, Schneider S, Schmucker K, Petruschke I, Kramer TS, Hanke R (2018). Antibiotic prescribing behavior among general practitioners—a questionnaire-based study in Germany. BMC Infect Dis.

[51] Rochford C, Sridhar D, Woods N, Saleh Z, Hartenstein L, Ahlawat H, Whiting E, Dybul M, Cars O, Goosby E, Cassels A (2018). Global governance of antimicrobial resistance. Lancet..

[52] Antao EM, Wagner-Ahlfs C (2018). Antibiotic resistance: a challenge for society. Bundesgesundheitsblatt Gesundheitsforschung Gesundheitsschutz..

[53] Klein EY, Van Boeckel TP, Martinez EM, Pant S, Gandra S, Levin SA (2018). Global increase and geographic convergence in antibiotic consumption between 2000 and 2015. Proc Natl Acad Sci USA.

[54] Golkar Z, Bagasra O, Pace DG (2014). Bacteriophage therapy: a potential solution for the antibiotic resistance crisis. J Infect Dev Ctries..

[55] UC SanDiego Health (2017). Novel phage therapy saves patient with multidrug-resistant bacterial infection [press release].

[56] Mullin E. Edible CRISPR could replace antibiotics. MIT Technol Rev. 2017. https://www.technologyreview.com/s/604126/edible-crispr-could-replace-antibiotics/.

[57] Bikard D, Euler CW, Jiang W, Nussenzweig PM, Goldberg GW, Duportet X (2014). Exploiting CRISPR-–Cas nucleases to produce sequence-specific antimicrobials. Nat Biotechnol.

[58] Zipperer A, Konnerth MC, Laux C, Berscheid A, Janek D, Weidenmaier C (2016). Human commensals producing a novel antibiotic impair pathogen colonization. Nature.

[59] Geldart KG, Kommineni S, Forbes M, Hayward M, Dunny GM, Salzman NH (2018). Engineered *E. coli* Nissle 1917 for the reduction of vancomycin-resistant Enterococcus in the intestinal tract. Bioeng Transl Med..

[60] Thaiss CA, Levy M, Grosheva I, Zheng D, Soffer E, Blacher E (2018). Hyperglycemia drives intestinal barrier dysfunction and risk for enteric infection. Science.

[61] Chassaing B (2015). The intestinal microbiota helps shapping the adaptive immune response against viruses. Med Sci (Paris)..

[62] Mohajeri MH, Brummer RJM, Rastall RA, Weersma RK, Harmsen HJM, Faas M (2018). The role of the microbiome for human health: from basic science to clinical applications. Eur J Nutr..

[63] Petrof EO, Gloor GB, Vanner SJ, Weese SJ, Carter D, Daigneault MC (2013). Stool substitute transplant therapy for the eradication of *Clostridium difficile* infection: ‘RePOOPulating’ the gut. Microbiome..

